# Socio-economic and socio-demographic determinants of diet diversity among rural pregnant women from Pune, India

**DOI:** 10.1186/s40795-022-00547-2

**Published:** 2022-07-05

**Authors:** Devaki Gokhale, Shobha Rao

**Affiliations:** 1 Symbiosis Institute of Health Sciences, Symbiosis International (Deemed University), Maharashtra, 412115 India; 2 Society for Initiatives in Nutrition and Diseases, Pune Maharashtra, 411007 India

**Keywords:** Diet diversity, Pregnancy, Socioeconomic determinants, Socio-demographic determinants

## Abstract

**Background:**

Diet diversity signifies the nutrient adequacy of an individual and thus has gained widespread significance in recent times. In developing countries achieving maximum diet diversity, especially among pregnant women from rural areas is challenging although of great importance. However, to do so understanding the primary factors associated with diet diversity is important. This paper, therefore, assessed the socio-demographic and socio-economic determinants of diet diversity among rural pregnant women in India.

**Methods:**

The study consisted of a community-based prospective cohort of *n* = 204 pregnant women attending primary healthcare centers (PHC) across 14 villages in Mulshi Taluka, Pune, Maharashtra, India. The data was collected using a structured questionnaire through a one-to-one interview method.

**Results:**

The prevalence of low, medium and high diet diversity was 56.4%, 33.3%, and 10.3% respectively. Minimum diversity in the diet was achieved among 73.5% of pregnant women. The mean diet diversity score (DDS) was 3.6 ± 1.3 with starchy staples being (100%) of commonly consumed foods. Young (< 20 years) women (OR = 5.2; CI:1.9- 13.8), housewives (OR = 3; CI:1.4–6.7), husbands working as skilled laborers (OR = 2.5; CI:1.2–5.5) were at significant risk of having low diet diversity scores. Whereas, those living in a joint family (OR = 0.3; CI:0.1–0.6), not owning a house (OR = 0.5; CI:0.2–0.9), and having a poor income (OR = 1.9; CI: 0.9- 3.7) were less likely to have low diet diversity.

**Conclusion:**

Socio-economic and demographic factors (maternal age, mother's occupation, and husband's occupation) influenced the diet diversity among pregnant women. Monotonous diets are commonly seen in developing countries, especially in rural areas which can be a risk factor for poor nutrient adequacy and health of pregnant women. Policies and programs about these determinants of diet diversity should be enacted to replace the poor quality diets to ensure improved diet diversity and nutrient adequacy.

## Background

The burden of maternal and child undernutrition in low and middle-income countries continues to be pervasive resulting in almost 3.5 million deaths [1]. Poor preconception nutrition, as well as nutrition during pregnancy, have both been associated with adverse pregnancy outcomes including intrauterine growth retardation [2] risk of low birth weight [3], stunting [4], and neonatal deaths globally [5]. Maternal undernutrition has been considered to be a predisposing factor for maternal mortality, as well as morbidity [6]. These studies provide evidence that the significance of maternal nutrition for better fetal outcomes and maternal and neonatal survival. Maternal diets thereby are important for improving the nutritional status and preventing poor pregnancy outcomes.

Diet diversification defined as an increase in consumption of various food groups capable of ensuring adequate nutrient intake [7] is one such avenue for improving the nutritional intake during pregnancy and avoiding the risk of poor pregnancy outcomes. Unfortunately, the diets of pregnant women in low- and middle-income countries (LMICs) are predominantly monotonous and plant-based with minimum consumption of micronutrient-dense non-vegetarian foods, fruits, and vegetables [8]. Lack of adequate resources and poor access are the primary reasons for poor diversified diets especially, among rural populations [9]. Thus, pregnant women in these countries face a challenge in meeting the increased nutrient needs during the most important physiological stage of life wherein the nutrient demands are higher. Furthermore, studies undertaken in developing countries indicate undernutrition [10], anemia [11], and poor health attributed to poor dietary intake.

In India, the triple burden of malnutrition continues and the micronutrient deficiencies have remained stagnant over the years [12]. Anemia in India is estimated to be higher as against other developing countries [13]. Also, anemia due to a lack of appropriate diet is the second leading cause of maternal deaths in the country [14]. Hence, in a developing country such as India, diversification of diets for rural communities is imperative. Additionally, it is important to identify the associated factors with inadequate diet diversity. Despite the importance given to diversification of diets, especially among developing countries in India, there is no study reporting the factors associated with inadequate diet diversity among vulnerable populations such as pregnant women from rural areas. Therefore, the objective of the study was to identify the potential determinants associated with dietary diversity among pregnant women. This study would thereby help formulation of policies to promote a diversified diet and enhance the nutrient intake among pregnant women.

## Methods

### Study setting and participants

A community-based prospective cohort of 204 pregnant women visiting the Primary Health Centre (PHC) from across 14 villages of Mulshi Taluka, Pune, Maharashtra, India was recruited for the study. These women were registered for the antenatal services provided by the PHC and used to visit once a month for a routine checkup. A comprehensive sample size of 196 was determined considering a prevalence of LBW of 30%, allowable error of 7%, and level of significance of 5% with 15% consideration for loss to follow up. However, we considered 204 women who visited the PHC for an antenatal checkup.

### Inclusion and exclusion criteria

All pregnant women (18- 25 years of age) who voluntarily consented to participate in the study and residents of Mulshi Taluka were included in the study. The pregnancy status was confirmed by a self-reported date of last menstrual period (LMP) in the healthcare record as well as using the USG reports available at the PHC. Multiple pregnancies, abortions, termination of pregnancies, and cases with congenital anomalies or chronic illness were excluded from the study considering the impact these conditions may have on the food intake of the pregnant women.

### Research tool

A questionnaire was designed with the reference of a research tool designed by the National Family Health Survey (NFHS-2015) Government of India. Modifications in the questionnaire were made considering community specifics obtained from various focus group discussions conducted by the researcher among pregnant women, community stakeholders, and direct family members. The questionnaire consisted of various questions to identify their socio-economic status and demographic details which included closed-ended questions on age, level of education, occupation of the mother, husband, type of family, monthly income, and ownership of the house. It was field-tested and amended after a pilot study to suit the study objectives and was administered by the researcher. The diet diversity questionnaire as prescribed by FAO was utilized for the identification of diet diversity scores (DDS) among pregnant women [15]. A 24-h diet recall was used for the assessment of DDS in pregnant women. The precaution was taken to include data on the actual consumption of foods daily. Days of fasting and other special festive occasions were avoided to capture the routine dietary intakes. The pregnant women were asked to recall all foods eaten and beverages consumed over the reference period of 24 h before the scheduled interview. As per the FAO [15] guidelines, a total of 9 food groups were included these consisted of starchy staples, dark green leafy vegetables, vitamin A-rich fruits and vegetables, other fruits and vegetables, organ meat, meat and fish, eggs, legumes nuts and oilseeds, milk and milk products. A score of one was given to each food group consumed over the reference period. A maximum score obtained for each individual could be a score of nine. The questionnaire was filled out by the researcher through one-to-one interviews with pregnant women.

### Outcome variables

#### Diet diversity score

The total number of food groups consumed out of a total of nine food groups by pregnant women.

#### Minimum diet diversity score

Intake of at least five food groups out of the nine food groups considered in the study within 24 h before the one-on-one scheduled interview.

#### Low diet diversity score

Intake of less than and equal to three food groups out of the nine food groups considered in the study within 24 h before the one-on-one scheduled interview.

#### Medium diet diversity score

Intake of four to five food groups out of the nine food groups considered in the study within 24 h* before the one-on-one scheduled interview.*

#### High diet diversity score

Intake of more than and equal to six food groups out of the nine food groups considered in the study within 24 h before the one-on-one scheduled interview.

### Statistical analysis

All the socio-demographic, socio-economic, and dietary data reported from the questionnaire were entered and verified by the researcher. Analysis was performed using the statistical package for Social Sciences (SPSS) (version 20; SPSS Inc., Chicago. IL, USA). The 9 food groups as per the FAO guideline were categorized as per the operational definitions mentioned earlier into low, medium, and high diet diversity scores. Various variables under the study were analyzed using descriptive statistics such as mean and standard deviation. Univariate regression analysis was used to identify the risk of low diet diversity due to various dependent variables such as maternal age, maternal education, maternal occupation, husband’s occupation, family income, family type, and ownership of the house. These variables were selected based on the National family health survey (NFHS) survey questionnaire for measuring socio-economic and demographic characteristics. A *p*-value of < 0.05 was considered to be significant.

## Results

### Socio-demographic and economic characteristics of pregnant women

A total of 204 pregnant women participated in this study with a mean age of 21.5 ± 2.1 years and their mean gestational age was 12 ± 1 week at the time of data collection. The majority of women (60.5%) were in the age group of 20 to 24 years. Most (43.1%) of the pregnant women were educated up to grade 10. More than half (57.8%) of the pregnant women were housewives with almost the remaining 42.1% engaged in labor-intensive activities such as farming or as a laborer. On the other hand, all husbands were employed either as a farmer (20.1%), skilled laborers (20.1%), or were in service (59.8%) these included jobs such as a clerk, and driver which was a salaried type of employment. Educational status and type of occupation determine the income of the family, this study noted a monthly family income of 8000 to 10,000 INR for the majority (40.7%) of the participants.

Notably, most (65.7%) of the participants did not own a house and were renting in the villages. The financial capacity of those paying rentals every month was better than those owning the house. In regards to the family characteristics, 62.7% stayed in a nuclear family which included up to 4 individuals in one family whereas 37.3% stayed in a joint family with more than 4 and up to 6 family members (Table 1).

**Table 1:** Socio-demographic and socio-economic characteristics of pregnant women

Variable	N (%)
**Maternal age**	
< 20	37 (18.1)
20–24	124 (60.8)^a^
> 24	43 (21.1)
**Maternal education**	
5^th^ grade	31 (15.2)
7^th^ grade	39 (19.1)
10^th^ grade	88 (43.1)*a*
12^th^ grade	46 (22.5)
**Maternal Occupation**	
Housewife	118(57.8)^a^
Farming	50(24.5)
Laborer	36(17.6)
**Husbands Occupation**	
Farmer	41 (20.1)
Skilled workers	41 (20.1)
Employed (self/service)	122 (59.8)^a^
**Family Type**	
Joint	76 (37.3)
Nuclear	128 (62.7)^a^
**Monthly Income**	
< 8000	58 (28.4)
8000–10,000	83 (40.7)^a^
> 10,000	63 (30.9)
**Ownership of House**	
No	134 (65.7)^a^
Yes	70 (34.3)

^a^ Majority of participants

### Consumption of foods by participants based on food groups

It was observed that starchy staples were predominantly consumed by all (100%) pregnant women followed by legumes, nuts, and oilseeds (81.4%) in their daily diet. This represents a typical Indian rural household diet comprising the cheapest sources of cereals in addition to a legume or nuts and oilseeds. Notably, foods of animal origin organ meat (7.8%), meat and fish (8.8%) as well as eggs (17.6%) were the least consumed by the pregnant women. (Table 2).

**Table 2:** Food groups consumed by pregnant women in the previous 24 h

Food Group	*n* = 204	%
Starchy staples	204	100
Dark green leafy vegetables	96	47.1
Vitamin A-rich fruits and vegetables	40	19.6
Other fruits and vegetables	107	54.5
Organ meat	16	7.8
Meat and Fish	18	8.8
Eggs	36	17.6
Legumes, nuts, and oilseeds	166	81.4
Milk and milk products	50	24.5

### Diet diversity of pregnant women

Out of 9 food groups, the study found that the mean DDS was 3.6 ± 1.3 with scores ranging from 3 to 9 food groups. Based on the categories developed, most respondents (56.1%) were in a low diet diversity group. The minimum diet diversity of ≥ 5 food groups were not achieved by more than one quarter (26.5%) of the respondents (Table 3).

**Table 3:** Diet diversity among rural pregnant women

Women’s diet diversity group	*n* = 204	%
Lowest (≤ 3 food groups)	115	56.4
Medium (4–5 food groups)	68	33.3
High (≥ 6 food groups)	21	10.3
Minimum diet diversity (≥ 5 food groups)	150	73.5

### Diet diversity categories and their association with the socio-economic and socio-demographic factors

The association between three diet diversity categories with socio-economic and socio-demographic factors was tested using univariate analysis. It was observed that the majority (75.7%) of pregnant women with a maternal age of < 20 years, mothers being housewives (63.6%), staying in a joint family (72.4%) with a monthly income of 8000–10,000INR (63.9%) were significantly associated with the low (< 3) diet diversity score. Further, it was also observed that a high diet diversity score (> 6) was reported in 75.7% of mothers above 24 years of age, 13% of mothers with an increased level of education above 10th grade, and 12.3% of husbands with salaried employment (Table 4).

**Table 4:** Diet diversity categories by socioeconomic and socio-demographic variables of rural pregnant women

Variables	n(%)	Low Diet Diversity (≤ 3 food groups) n(%)	Medium Diet Diversity (4–5 food groups) n(%)	High Diet Diversity (≥ 6 food groups) n(%)	*P* value
**Maternal Age**					
< 20	37(18.1)	28(75.7)	9(24.3)	0(0)	0.007^a^
20–24	124(60.8)	71(57.3)	39(31.5)	14(11.3)	
> 24	43(21.1)	16(37.2)	20(46.5)	7(16.3)	
**Mothers Education**					
5^th^ grade	31(15.2)	18(58.1)	11(35.5)	12(6.5)	0.817
7^th^ grade	39(19.1)	21(53.8)	15(38.5)	3(7.7)	
10^th^ grade	88(43.1)	53(60.2)	25(28.4)	10(11.4)	
12^th^ grade	46(22.5)	23(50)	17(37)	6(13)	
**Mothers Occupation**					
Housewife	118(57.8)	75 (63.6)	31(26.3)	12(10.2)	0.012^a^
Farming	50(24.5)	27(54)	21(42)	2(4)	
Laborer	36(17.6)	13(36.1)	16(44.4)	7(19.4)	
**Husbands Occupation**					
Farmer	41(20.1)	27 (65.9)	10 (24.4)	4(9.8)	0.075
Skilled workers	41(20.1)	29(70.7)	10(24.4)	2(4.9)	
Employed	122(59.8)	59(48.4)	48(39.3)	15(12.3)	
**Family Type**					
Joint	76(37.3)	55(72.4)	13(17.1)	8(10.5)	0.001^a^
Nuclear	128(62.7)	60(46.9)	55(43)	13(10.2)	
**Monthly Income**					
< 8000	58(28.4)	32(55.2)	15(25.9)	11(19)	0.025^a^
8000–10,000	83(40.7)	53(63.9)	26(31.3)	4(4.8)	
> 10,000	63(30.9)	30(47.6)	27(42.9)	6(9.5)	
**Ownership of House**					
No	134 (65.7)	68(50.7)	51(38.1)	15(11.2)	0.070
Yes	70 (34.3)	47(67.1)	17(24.3)	6(8.6)	

^a^ Statistically significant association between dietary diversity categories and the variable at *p* ≤ 0.05

### Associated factors of diet diversity among pregnant women

The association between various dependent and independent factors was explored using multiple logistic regression. It was observed that mothers with age < 20 years (OR = 5.2; CI: 1.9–13.8) and those between 20–24 years (OR = 2.2; CI: 1.4–4.6) had 5 times and 2 times greater odds of having low diet diversity respectively. The employment status of pregnant women and their husbands revealed that those women who were unemployed (housewives) (OR = 3; CI: 1.4–6.7) had a 3 times higher risk of having low diet diversity. Pregnant women whose husbands did not have a salaried job working as skilled laborers (OR = 2.5; CI: 1.2–5.5) had twice the risk of having low diet diversity. Those with a monthly family income of 8000 to 10,000 INR (OR = 1.9.; CI: 0.9–3.7) had the highest risk for low diet diversity. However, those staying in a joint family (OR = 0.3.; CI: 0.1–0.6), as well as those with no ownership of a house (OR = 0.5; CI: 0.2–0.9), had the lowest risk for low diet diversity (Table 5).

**Table 5:** Univariate logistic regression showing socio-economic and socio-demographic factors associated with low (≤ 3) diet diversity among rural pregnant women

Variables	n(%)	OR (95%CI)	*p* value
**Maternal Age**			
< 20	37(18.1)	5.2(1.9–13.8)	0.001^a^
20–24	124(60.8)	2.2(1.1–4.6)	0.025^a^
> 24	43(21.1)	1	
**Mothers Education**			
5^th^ grade	31(15.2)	1.3(0.5–3.4)	0.487
7^th^ grade	39(19.1)	1.1(0.4–2.7)	0.724
10^th^ grade	88(43.1)	1.5(0.7–3.1)	0.258
12^th^ grade	46(22.5)	1	
**Mothers Occupation**			
Housewife	118(57.8)	3(1.4–6.7)	0.004^a^
Farming	50(24.5)	2(0.8–5)	0.103
Laborer	36(17.6)	1	
**Husbands Occupation**			
Farmer	41(20.1)	2 (0.9–4.3)	0.055^a^
Skilled workers	41(20.1)	2.5(1.2–5.5)	0.015^a^
Employed	122(59.8)	1	
**Family Type**			
Joint	76(37.3)	0.3(0.1–0.6)	0.001^a^
Nuclear	128(62.7)	1	
**Monthly Income**			
< 8000	58(28.4)	1.3 (0.6–2.2)	0.407
8000–10,000	83(40.7)	1.9(0.9–3.7)	0.051^a^
> 10,000	63(30.9)	1	
**Ownership of House**			
No	134 (65.7)	0.5(0.2–0.9)	0.026^a^
Yes	70 (34.3)	1	

*OR* Odds ratio, *CI* Confidence interval

^a^ Statistically significant association between low dietary diversity and the variable at *p* < 0.05

## Discussion

An increasing number of studies from India and developing countries have documented the role of poor maternal malnutrition resulting from a series of factors deep-rooted in poverty primarily due to socio-demographic and socio-economic inequalities [16–18]. However, despite the importance given to diet diversity as one of the factors for maternal malnutrition the role of various socio-demographic and economic determinants of inadequate diet diversity are largely unexplored in India. Hence, the present study investigated the determinants of diet diversity among pregnant women from Mulshi Taluka, Pune, Maharashtra, India. The study was conducted among pregnant women attending the primary healthcare center (PHCs) across 14 villages.

The mean DDS was 3.6 ± 1.3 with one-fourth (26.5%) of the population not being able to achieve a minimum (≥ 5) diet diversity score. Studies from developing countries such as Kenya [19] report a smaller percentage (15%) of pregnant women not being able to attain the minimum diet diversity score (≥ 5). On the contrary, a study from Ethiopia [20] report a higher percentage (74.6%) being unable to achieve the minimum diet diversity score (≥ 5).

Overall, the majority (56.1%) of pregnant women had a low (< 3) diet diversity score with only 10% having a high diet diversity score of ≥ 6 food groups. Interestingly, it has been reported by various studies in developing countries of Bangladesh [21] and Tanzania [22] that diets in these countries are predominantly based on starchy staples. The findings from the present study report that 100% of pregnant populations consume food items from the starchy staples group including grain and grain products daily with only a few consuming (8.9%) meat and fish. This substantiates the findings from the previous studies indicating the predominance of monotonous diets, especially among rural populations [19, 22]. Apart from the aforementioned food groups legumes, nuts, and oilseeds (81.4%) were consumed majorly in form of *dal* preparation (boiled and seasoned legumes) as well as groundnuts and oilseeds as *chatni* (usually consumed as a side accompaniment along with Indian bread) which are components of a typical Indian meal. Vegetables and fruits (54.5%), and dark green leafy vegetables (47.1%) were consumed by close to only half of the women in their daily diet. Similarly, milk and milk products being a very important source of calcium and proteins for vegetarians was consumed poorly by only 24.5% of pregnant women. This could be attributed partly to socio-economic factors of poor affordability and poor awareness among pregnant women and partly by the socio-demographic factors of young age and family type.

Regarding the association between various diet diversity categories and socio-economic and demographic factors of level of maternal education, husbands' occupation and ownership of the house were not significantly associated. This was contrary to the previous findings which suggest improvement in education levels [23–25]. However, it was observed that with an improvement and an increase in maternal age, salaried employment among mothers, and monthly family income the diversity in the diets improved significantly. Other studies from developing countries too showed a similar association between diet diversity and socio-demographic and economic variables [20, 26, 27]. Further, when we looked at the risk of low diet diversity (≤ 3) we found that lower age (< 24 years), housewives, husbands' occupation (non-salaried) as skilled laborers, and poor income were significantly associated. In rural areas, the indicators of socio-economic characteristics are often measured in terms of proxy indicators of household assets, ownership of land and houses, and livestock apart from the monthly income of the family. The findings from this study indicated that those with a family income of only 8000-1000INR had the highest risk for low diet diversity. These results were in tandem with previously reported studies which indicate poor socio-economic conditions related to lower diet diversity [28–30]. Similarly, for the socio-demographic variables, often women in rural areas are married at a younger age with lower levels of education [31]. This invariably translates into lower chances of employment and most of them end up being housewives with a lack of awareness about diet and nutrition. For those who stay in nuclear families where the family size is less than four members access to food primarily is a challenge. Since the sole breadwinner is just a single family member there is poor affordability and diet adequacy. In such a situation where the bare minimum requirements of daily diets are not met, the diet diversity is still secondary and far-fetched. These findings indicate the impact of poor socio-demography and poor socio-economic characteristics which imposes a risk for lower diversification of diets.

We would like to indicate that firstly there can be multiple determinants associated with diet diversity, which have not been included in the current study. Yet, the overwhelming importance of socio-demographic and economic factors on diet diversity especially in rural communities of India cannot be denied. Secondly, we did not assess the quantity of food which needs to be studied further. However, our analysis identified the determinants of poor diet quality in terms of low diet diversity scores. The small sample size and the cross-sectional design may also be subject to confounding in this study.

## Conclusion

The study demonstrated that there is an association between socio-demographic and socio-economic determinants with the diet diversity among rural pregnant women. In the light of the explicit findings of this study, there is a need for focusing on the viable interventions and programs that help in improving and sustaining the diet diversity among pregnant women. The critical role of factors such as maternal age, education, occupation, husband’s occupation, family type, monthly income, and ownership of the house in the attainment of adequate diet diversity is inevitable.

## Data Availability

The datasets used and analysed for the present study will be available from the corresponding author on reasonable request.

## Abbreviations

OR: Odds ratio
CI: Confidence interval
DDS: Diet diversity score
FAO: Food and agriculture organization
